# Electrochemical characterization and chronic stability of Utah electrode arrays for intracortical microstimulation

**DOI:** 10.3389/fnins.2026.1789112

**Published:** 2026-03-30

**Authors:** Christopher K. Nguyen, Brandon S. Sturgill, Balaji Srikanthan, Behnoush Dousti, Madhav Bhatt, Mahasty Khajehzadeh, Soham Mangarolia, Nashita Hasan, Ana G. Hernandez-Reynoso, Sandeep Negi, Stuart F. Cogan

**Affiliations:** 1 Department of Bioengineering, The University of Texas at Dallas, Richardson, TX, United States; 2 Blackrock Neurotech, Salt Lake City, UT, United States; 3 Department of Biomedical Engineering, Case School of Engineering School of Medicine, Case Western Reserve University, Cleveland, OH, United States

**Keywords:** amorphous silicon carbide, charge density, cyclic voltammetry, encapsulation, neural stimulation, Parylene-C, sputtered iridium oxide, Utah electrode array

## Abstract

This study presents methods for evaluating the long-term electrochemical stability of Utah electrode arrays (UEAs) encapsulated with amorphous silicon carbide (a-SiC) or Parylene-C for intracortical microstimulation (ICMS). UEAs were implanted in rat motor cortex and monitored for 25 weeks using electrochemical impedance spectroscopy (EIS) to observe device impedance, cyclic voltammetry (CV) to obtain cathodic charge-storage capacity (*Q*_stor,c_), and voltage transient (VT) measurements to derive maximum charge-injection capacity (*Q*_inj_). Observed shifts in the open-circuit potential of the return electrode during stimulation, from in vitro to *in vivo* conditions, highlight the need to adjust potential limits when using a quasi-reference electrode. Both encapsulation materials exhibited stable impedance and maximum (*Q*_inj_) trends *in vivo*, becoming similar after approximately 16 weeks post-implant. High-scan rate CV (up to 500,000 mV/s) was used to assess similarities between *Q*_stor,c_ and maximum *Q*_inj_ and determine if it can be used to estimate the maximum *Q*_inj_. These methods can be used to evaluate the encapsulation and stimulation properties of chronically implanted neural electrodes.

## 1 Introduction

Neuromodulation has advanced significantly, enabling the restoration of sensory and motor function, the treatment of neurological disorders, and the development of sophisticated neural interface systems (Szostak et al., 2017). A critical component of these technologies is the electrode array, which establishes a direct interface with the nervous system. Among the most widely used microelectrode arrays (MEAs) for intracortical microstimulation (ICMS) are the Utah electrode arrays (UEAs), which have also been used for neural recording. These arrays, consisting of a grid of penetrating microelectrodes, allow for precise stimulation and recording of neural activity at the single-neuron level (Branner and Normann, 2000). This makes UEAs particularly suitable for neural applications such as motor rehabilitation (Hatsopoulos and Donoghue, 2009; Hughes et al., 2021), and cortical mapping (Flesher et al., 2016).

Despite their utility, the long-term stability of neural electrodes remains a significant challenge. Stimulation-induced stress and the foreign body response (FBR) can lead to degradation of both the electrodes and surrounding tissue (McCreery et al., 1995; Cogan et al., 2004a,^2016^; Merrill et al., 2005; Butterwick et al., 2007; Vatsyayan et al., 2021). This degradation often manifests as increased impedance, reduced charge-injection, and mechanical failure (Rousche and Normann, 1998). Although studies have reported chronic impedance trends (Abbott et al., 2024) or fixed charge-per-phase testing (Black et al., 2018; Joshi-Imre et al., 2019), fewer investigations have systematically tracked how maximum charge-injection capacity (*Q*_inj_) evolves over time during *in vivo* implantation. Furthermore, the performance of the electrode-tissue interface depends on the electrode materials’ electrochemical properties and their stability over prolonged periods. Improving the encapsulation materials is essential for extending the long-term use of neural implants.

Encapsulation materials protect MEAs from the surrounding biological environment while preserving device functionality. Parylene-C is a polymer widely used for medical device encapsulation, offering good barrier properties, chemical stability, and ease of fabrication (Schmidt et al., 1988). However, the long-term durability of Parylene-C can be limited *in vivo*, as cracks, delamination, and blistering have been reported after chronic implantation (Loeb et al., 1977; Schmidt et al., 1988; Barrese et al., 2016). In contrast, amorphous silicon carbide (a-SiC) has emerged as a promising alternative due to its mechanical durability, corrosion resistance, and electrochemical stability (Cogan et al., 2003; Nguyen et al., 2021). While a-SiC has shown promise in chronic studies using rodent models (Joshi-Imre et al., 2019; Usoro et al., 2019; Abbott et al., 2024), further investigation is required to evaluate longitudinal electrode charge-injection behavior and changes over time, particularly under chronic stimulation conditions. The aim of this paper is to determine whether a-SiC maintains stable electrochemical stability comparable to Parylene-C during long-term implantation.

Electrochemical techniques, such as electrochemical impedance spectroscopy (EIS) and cyclic voltammetry (CV), are widely employed to assess neural electrode performance (Cogan, 2008). EIS provides information on electrode characteristics across frequencies, reflecting the electrode’s ability to conduct current at the tissue interface. CV, on the other hand, assesses the electrode material’s features and cathodic charge-storage capacity (*Q*_stor,c_), a parameter that has some relation to charge-injection capability (Kelliher and Rose, 1987; Frederick et al., 2020). However, these methods do not directly determine changes in stimulation electrode properties, such as maximum *Q*_inj_, during long-term implantation.

This study presents methods for evaluating the electrochemical performance of UEAs encapsulated with Parylene-C or a-SiC with emphasis on longitudinal determination of maximum *Q*_inj_ rather than fixed-charge testing. By employing high-sweep CVs (up to 500,000 mV/s)—exceeding typical CV sweep rates for determining *Q*_stor,c_ and capturing fast electrochemical dynamics—and automated voltage transient (VT) measurements, this work reports chronic maximum *Q*_inj_ tracking over a 25-week implantation period to quantify stimulation electrode performance. Previous *in vivo* studies have predominantly focused on fixed *Q*_inj_ for chronic stability (Meijs et al., 2015; Black et al., 2018; Joshi-Imre et al., 2019), acute electrode characterization (Brunton et al., 2015), tissue damage thresholds (Cogan et al., 2004a; McCreery et al., 2010; Vatsyayan et al., 2021), or psychophysical thresholds (Smith et al., 2023). This paper instead focuses on dynamically tracking the maximum *Q*_inj_, enabling a comparison of Parylene-C and a-SiC devices both initially in phosphate-buffered saline (PBS) and subsequently in rat motor cortex models. By examining how encapsulation affects electrochemical behavior over time, this work provides a method for assessing long-term electrochemical stability and charge-injection performance. The primary objective is to evaluate longitudinal electrochemical and stimulation performance of two UEA device implementations—one encapsulated with a-SiC and one with Parylene-C—under chronic implantation conditions.

## 2 Materials and methods

### 2.1 Device fabrication

UEAs were fabricated with 3-μm Parylene-C encapsulation and sputtered iridium oxide film (SIROF) electrodes at Blackrock Neurotech (BRN) (Bhandari et al., 2010). Additional UEAs with a-SiC encapsulation were produced at the University of Texas at Dallas (UTD) in a 10 × 10 configuration (Joshi-Imre et al., 2019). The arrays were inspected using a Zeiss SIGMA 500VP scanning electron microscope (Carl Zeiss AG, GE) at UTD. Fabrication of the a-SiC UEAs began with RCA cleaning to remove particulates and debris to prepare the doped-Si substrate for a-SiC deposition (Nguyen et al., 2022). Plasma-enhanced chemical vapor deposition (PECVD) was performed on a Plasma-Therm Vision 310 Series system (Plasma-Therm, LLC, United States) using deposition parameters of 300°C, 0.8 Torr, 200-W RF power (density of 0.36 W/cm^2^), 600 sccm of 2% SiH_4_ with Ar carrier gas, 36 sccm CH_4_, and 164 sccm Ar (Nguyen et al., 2024).

**FIGURE 1:**
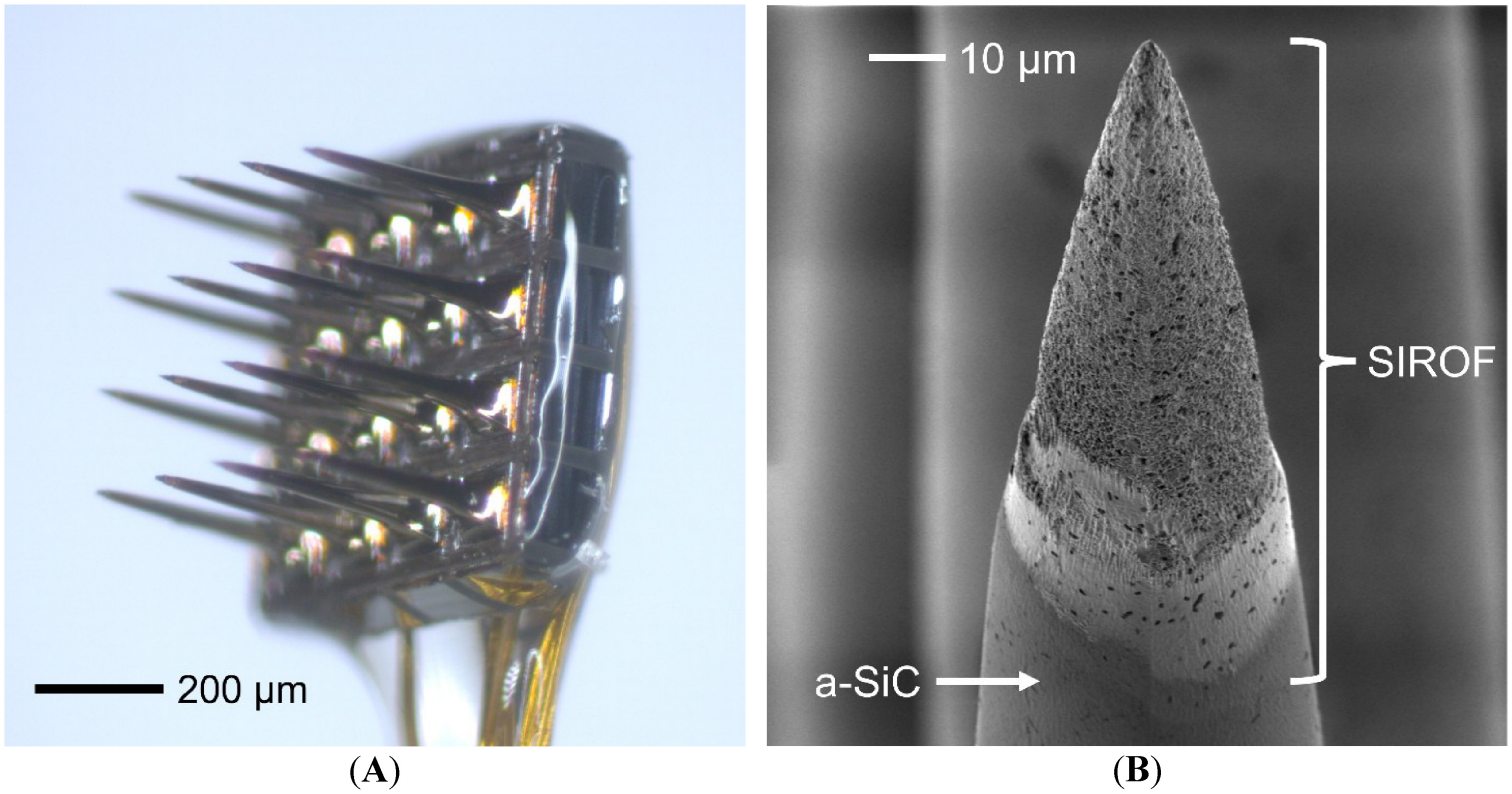
Image of assembled UEA with a-SiC encapsulation and UTD SIROF electrodes as received from BRN. **(A)** Optical image overview of the 4 × 4 UEA. **(B)** Scanning electron micrograph of an electrode tip with approximately 5,000 μm^2^ SIROF.

The tips of UEAs with a-SiC encapsulation were exposed as electrode sites by reactive ion etching (RIE) using a Trion Sirus-T2 system (Trion Technology, United States) with parameters of 0.1 Torr, 350 W (power density of 0.88 W/cm^2^), 3 sccm SF_6_, 7 sccm O_2_, and 2 sccm Ar for higher a-SiC/Si selectivity (Nguyen et al., 2022). The a-SiC arrays had SIROF deposited by DC magnetron sputtering with an AJA ATC 2200 system (AJA International Inc., United States) as an electroactive coating (Maeng et al., 2019). All arrays were diced into a 4 × 4 configuration, as shown in Figure 1A, and Au-wire bonded to an 18-pin Omnetics connector (Omnetics Connector Corporation, United States). The electrode’s geometric surface area (*A*_gs_) of both a-SiC and Parylene arrays is approximately 5,000 μm^2^, illustrated in Figure 1B.

### 2.2 Animal surgery

All animal handling, surgery, and experiments were carried out in accordance with the UTD Institutional Animal Care and Use Committee. The implantation protocol was adapted from previous UEA studies (Black et al., 2018; Joshi-Imre et al., 2019). Adult male Sprague-Dawley rats (Charles River Laboratories, United States) were used. Anesthesia was initiated with 3% isoflurane in 100% O_2_ at 500 mL/min on a Kent Scientific SomnoSuite system (Kent Scientific Corporation, United States) before being fixed on a Kopf model 940 stereotaxic frame (Kopf Instruments, United States). The isoflurane was reduced to 2–2.5% throughout the surgery. Dexamethasone (2 mg/kg) was injected subcutaneously between the shoulders to reduce inflammation, while lidocaine (20 mg/kg) was distributed subcutaneously along the scalp for analgesia. A 3 × 3-mm^2^ craniotomy window in the motor cortex region was centered approximately 2.5 mm rostral and 2.5 mm lateral (right) to bregma to provide adequate space for UEA placement. UEAs were implanted using a pneumatic insertion (8.3 m/s) with a Blackrock Electrode Inserter System (Blackrock Neurotech, United States) (Rousche and Normann, 1992). A 3D-printed adaptor for the tip of the inserter was designed to enable visual access and align the device with the craniotomy window (Nguyen et al., 2024).

### 2.3 Electrochemical experiments

**FIGURE 2:**
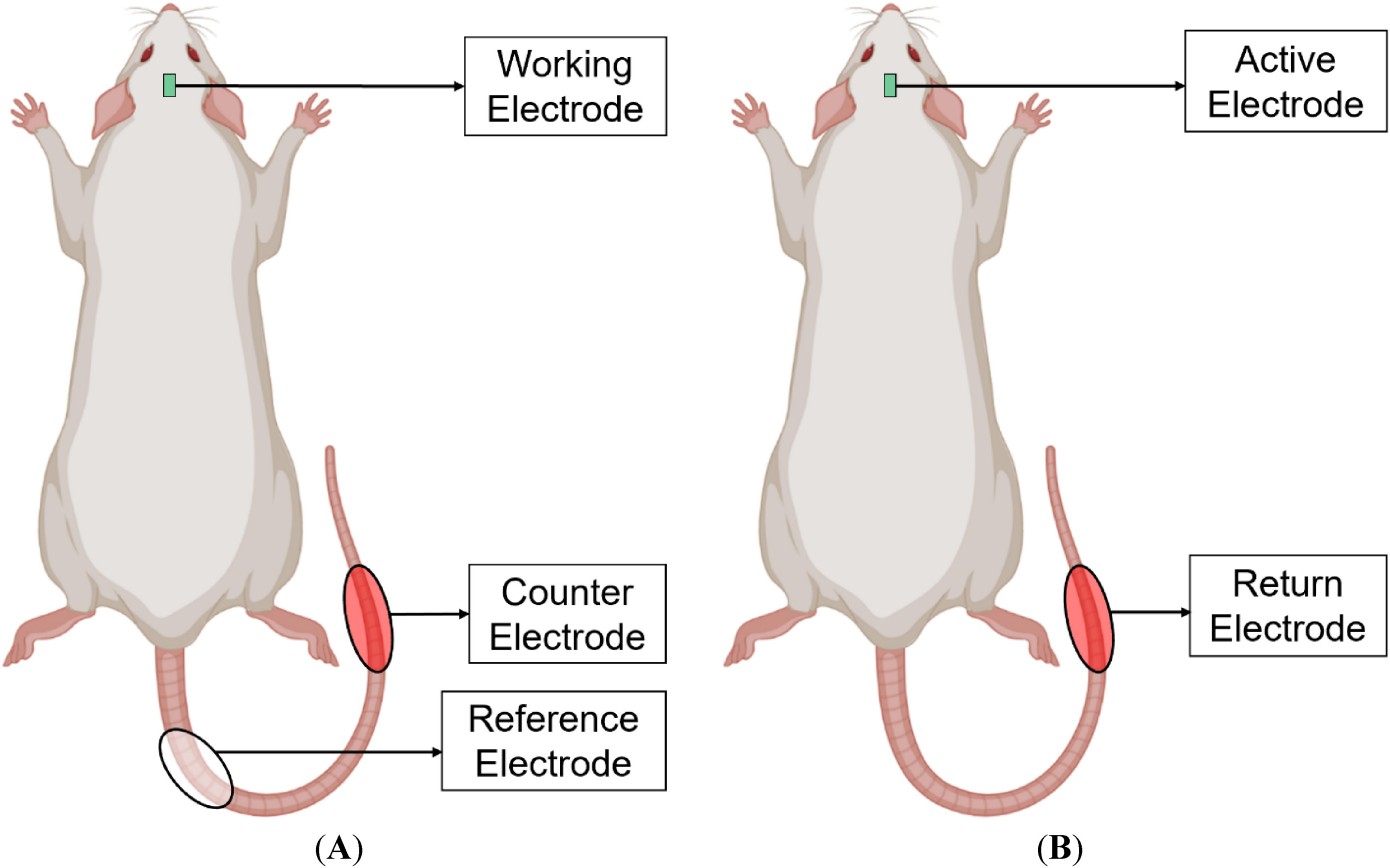
Experimental setup for rat model. **(A)** Three-electrode system for electrochemical measurements with a potentiostat and **(B)** two-electrode system for voltage transients with a microstimulator.

Electrochemical measurements were conducted using a Gamry Reference 600 potentiostat (Gamry Instruments, United States) in a three-electrode setup. *In vitro* measurements were made in phosphate-buffered saline (PBS) at 37°C. The *in vitro* reference electrode was a glass-bodied Ag|AgCl electrode with 3 M NaCl (Bioanalytical Systems Inc., United States), and the counter electrode was a large-area Pt wire (Bioanalytical Systems Inc., United States). The *in vivo* reference was a Ag|AgCl patch electrode (BIOPAC Systems Inc., United States) with electrode gel (BIOPAC Systems Inc., United States) wrapped around the basal end of the tail, and the counter was a PtIr (90/10) needle electrode (Technomed, NL) inserted at the distal end of the tail (see Figure 2A).

The equilibrium, or open-circuit, potential (*E*_oc_) of the electrode was measured briefly as the starting potential for EIS and CV. The first measurement conducted was EIS, ranging from 100 kHz to 1 Hz with a 10-mV RMS perturbation. Metrics include tracking changes at 1 Hz and 1 kHz over time. Analysis of high-frequency impedance was limited to 32 kHz to reduce contributions from instrumentation noise (Lutz et al., 2025). EIS was performed only once per week at the beginning of each session. The CV measurements were performed after EIS at scan rates (*v*_scan_) from 50 to 500,00 mV/s to investigate the cathodic charge-storage capacity (*Q*_stor,c_) dependence on sweep and any correlation with *Q*_inj_. Slow *v*_scan_ was used to monitor leakage development, while fast *v*_scan_ was used to assess electrode surface integrity. A high *v*_scan_ of 500,000 mV/s was used to determine whether *Q*_stor,c_ derived from fast CV approximates pulse-derived *Q*_inj_, thereby evaluating whether high-sweep CV can estimate stimulation-relevant charge densities when voltage transient setups are unavailable for maximum charge-injection capacity measurements. The 500,000 mV/s limit was selected to remain within the potentiostat’s resolution. All CV measurements were conducted with 10-mV potential steps, where tested sweeps used sampling periods of 0.2 S/s for 50 mV/s to 20 μs/S for 500,000 mV/s. These sampling periods remain within the Gamry Reference 600’s resolution of 3.33 μs/S. The CVs were measured over the water electrolysis window of SIROF, between a cathodic potential limit (*E*_lc_) of −0.6 versus Ag|AgCl and an anodic potential limit (*E*_la_) of 0.8 V versus Ag|AgCl (Cogan et al., 2004b). *Q*_stor,c_ was calculated from.


$$Q_{\mathrm{stor},c}=\frac{1}{A_{\mathrm{gs}}}\,\int_{0}^{t_{\mathrm{cycle}}}i_{c}\,\left(t\right)\,dt$$ (1)

Equation (1) calculates charge as a function of time over one cycle of CV (*t*_cycle_) at a time differential (d*t*)—the sampling period. Since the data are discrete, rather than continuous, the equivalent summation representation is used:


$$Q_{\mathrm{stor},c}=\frac{1}{A_{\mathrm{gs}}}\,\lim_{\Delta \,t\to 0}\overset{t_{\mathrm{cycle}}}{\underset{t=0}{\sum }}i_{c}\,\left[t\right]\,\Delta \,t$$ (2)

Equation (2) is the right-rule Riemann sum for the discrete integration of current over time—the summation of charge. The d*t* is determined by the fixed time step size (Δ*t*) with potential step size (Δ*E*) as:


$$\Delta \,t=\frac{\Delta \,E}{v_{\mathrm{scan}}}$$ (3)

The number of points in a single curve (*N*) is given by the following:


$$N=\frac{t_{\mathrm{cycle}}}{\Delta \,t}+1$$ (4)

Equation (2) is modified as a trapezoidal-rule Riemann sum for a more precise computation of *Q*_stor,c_:


$$Q_{\mathrm{stor},c}=\frac{1}{A_{\mathrm{gs}}}\,\overset{N-1}{\underset{n=1}{\sum }}\left(\frac{i_{c}\,\left[t_{n-1}\right]+i_{c}\,\left[t_{n}\right]}{2}\right)\,\Delta \,t$$ (5)

Equation (5) designates *t*_0_ as the first data point.

### 2.4 Microstimulation experiments

Following 48 h after electrochemical measurements (EIS and CV), microstimulation was performed to measure VTs. A custom script was developed to perform and monitor VT measurements and to determine charge-injection capacity (*Q*_inj_) automatically. A PlexStim stimulator system (Plexon Inc., United States) was used to deliver biphasic current pulses, record VT waveforms, and enable programmable control. The PlexStim was set to discharge mode, with active and return electrodes shorted together during the interpulse. The VT response and applied current waveforms were captured with a Tektronix TBS1164 oscilloscope (Tektronix Inc., United States). Stimulation utilized a two-electrode setup, where the PtIr needle electrode was used as the return electrode (serving as both reference and counter), as shown in Figure 2B. PtIr was chosen as a return electrode for its electrochemical stability under current pulsing (Dalrymple et al., 2020), as opposed to stainless steel, which often exhibits an unstable *E*_oc_ during long-term use (Sawada et al., 2024).

The script was developed in MATLAB R2021b (MathWorks Inc., United States) using the Instrument Control Toolbox and NI-VISA (National Instruments Corporation, United States) for Virtual Instrument Software Architecture (VISA). Current-controlled microstimulation was composed of a cathodic-first, biphasic, symmetric waveform with 200-μs phase width (*t*_ph_) and 100-μs interphase delay and delivered at 50 pps. ICMS was not administered outside the VT sessions for the animals. The charge-per-phase (*Q*_ph_) with the magnitude of current amplitude (*I*_stim_) is given below:


$$Q_{\mathrm{ph}}=\left|I_{\mathrm{stim}}\right|\cdot t_{\mathrm{ph}}$$ (6)

$$Q_{\mathrm{inj}}=\frac{Q_{\mathrm{ph}}}{A_{\mathrm{gs}}}$$ (7)

Equation (7) defines the charge-injection capacity (*Q*_inj_) as *Q*_ph_ per unit area. To determine the maximum *Q*_inj_, the script adjusted the *I*_stim_ based on how far away the maximum cathodic potential excursion (*E*_mc_) was from *E*_lc_. Pulsing stopped when *E*_mc_ reached *E*_lc_ within ± 20 mV. Each electrode measurement was completed sequentially. Because PtIr was used as a reference electrode, the water window was adjusted with the formula below:


$$E^{{}^{\prime }}=E-E_{\mathrm{oc}}$$ (8)

Equation (8) shifts a measured potential (*E*) by the return electrode *E*_oc_, measured against Ag|AgCl, to an adjusted potential (*E*′) which is now referenced to Ag|AgCl. In PBS, the Pt return electrode *E*_oc_ was measured to be 0.221 ± 0.027 V versus Ag|AgCl in PBS (*n* = 54, mean ± SD) such that (8) yields a new cathodic potential limit ($E_{\mathrm{lc}}^{\prime }$) of -0.8 V versus PtIr. However, in rat, *E*_oc_ was found to be -0.021 ± 0.033 V versus Ag|AgCl (*n* = 26, mean ± SD). Therefore, PtIr *E*_oc_ in rat is approximately equivalent to Ag|AgCl. For determining the maximum *Q*_inj_:


$$\max\,\left(Q_{\mathrm{inj}}\right)=\lim_{E_{\mathrm{mc}}\to E_{\mathrm{lc}}\pm 0.02}\left(Q_{\mathrm{inj}}\right)$$ (9)

**FIGURE 3:**
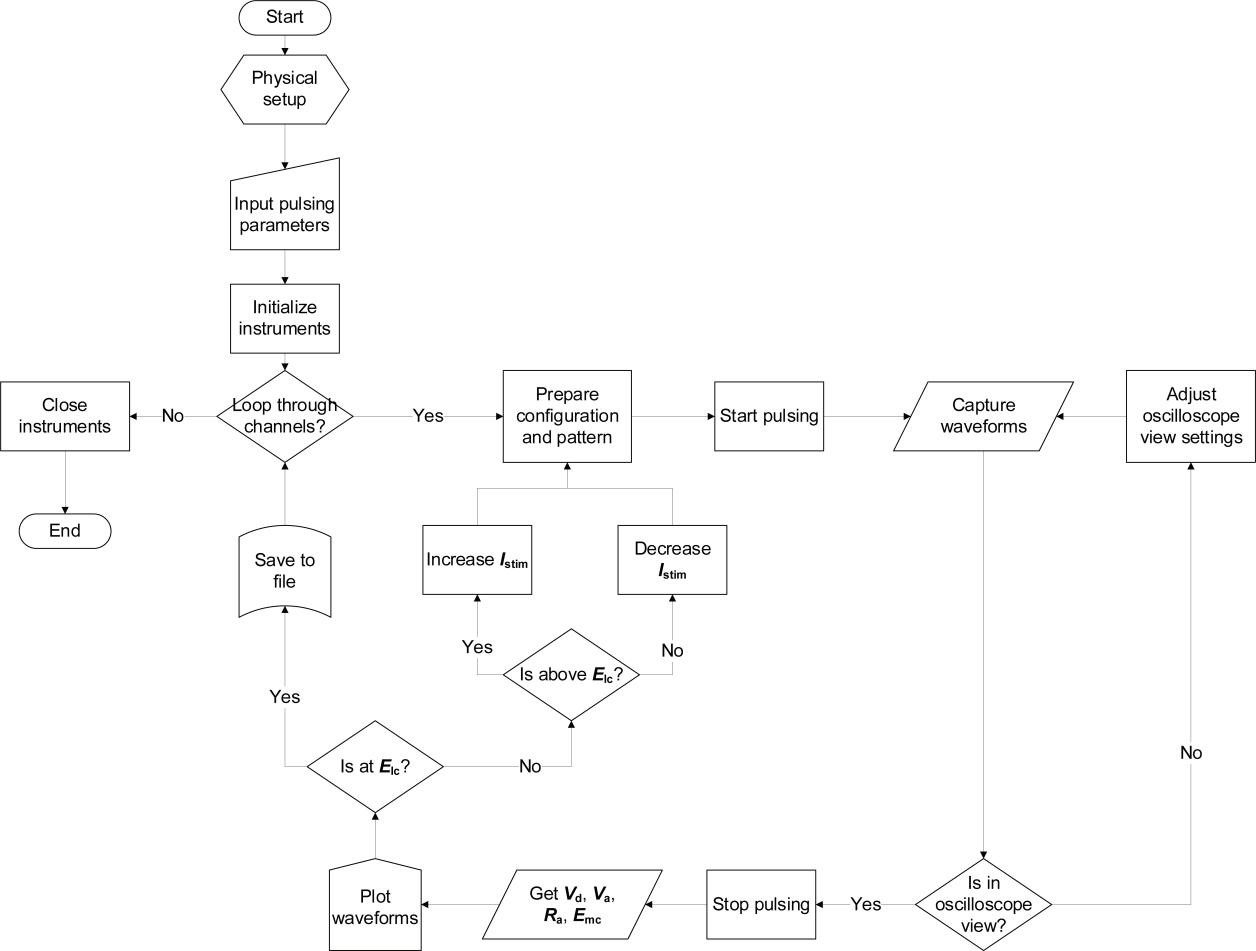
Flowchart for automated voltage transients with microstimulator and oscilloscope.

Equation (9) is the formula employed to determine the maximum *Q*_inj_ as *E*_mc_ reaches *E*_lc_ with a tolerance of ± 20 mV (Nguyen et al., 2024). The automated VT program sequentially tests each channel on an MEA by applying an adjustable *I*_stim_, evaluating *E*_mc_, and recording the associated metrics, as outlined in Figure 3. For each tested *I*_stim_ until maximum *Q*_inj_ could be found, pulsing was held until the oscilloscope could properly capture waveforms in window view—approximately 2 s. It should be noted that this duration is sufficient for VTs to reach steady-state. Due to starting and stopping process for each tested *I*_stim_ and variability between electrodes, total number of pulses or charge is difficult to estimate.

### 2.5 Statistical analysis

All statistical analyses and plots were generated in GraphPad Prism 10. Each UEA group (a-SiC with UTD SIROF and Parylene-C with BRN SIROF) consisted of 2 arrays (total of 4 arrays and animals) and 16 electrodes per array (32 electrodes per group). The figures reflect the number of active electrodes (*n*) throughout the study; a percentage yield was included, based on characterization in PBS. Because arrays represent the true biological replicates (*N* = 2 per group), the study was not powered to detect small between-group differences. Electrodes within a given array were therefore not treated as independent replicates for formal hypothesis testing.

Given the limited number of independent arrays and the non-linear, non-monotonic longitudinal trajectories observed across metrics, statistical analysis emphasized descriptive reporting (mean ± 95% confidence intervals and longitudinal trends) rather than formal hypothesis testing. Any timepoint-wise statistical comparisons were considered exploratory and interpreted cautiously.

## 3 Results

### 3.1 Electrochemical measurements

#### 3.1.1 Electrochemical impedance spectroscopy

**FIGURE 4:**
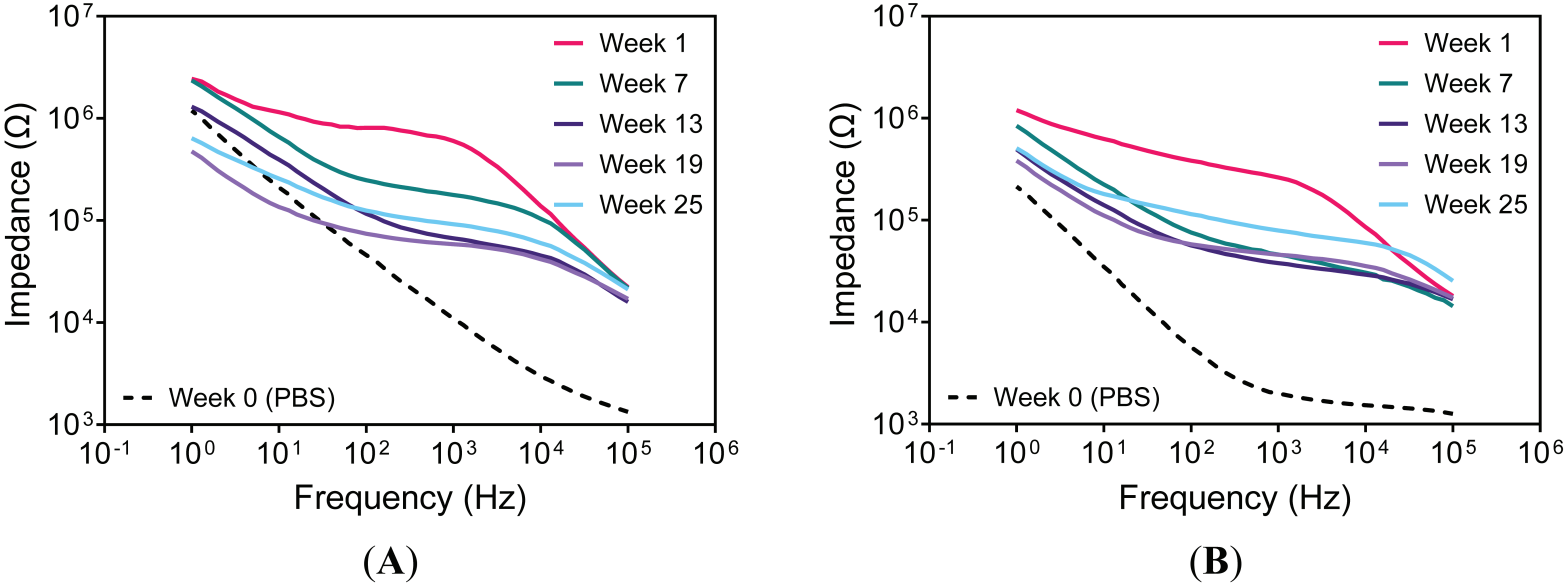
Representative impedance spectra of an electrode on a UEA. Devices with **(A)** a-SiC encapsulation and UTD SIROF or **(B)** Parylene-C encapsulation and BRN SIROF.

Although 1-kHz impedance is commonly used to evaluate recording electrodes, analyzing frequencies such as 1 Hz and 32 kHz can provide more information about device behavior. At 1 kHz, the impedance approximates the characteristic frequency of neuronal action potentials. On UEAs, reductions in 1-Hz impedance are suggestive of hydration of SIROF (Hackwood et al., 1981; Boretius and Stieglitz, 2013) or encapsulation failure near bond pads or the Au-wire bundle (Joshi-Imre et al., 2019). At higher frequencies ( > 10 kHz), the impedance may be influenced by surface changes at the electrode-tissue interface. As expected, noticeable shifts in EIS from PBS to *in vivo* conditions were observed for both a-SiC and Parylene-C encapsulated devices, as shown in Figure 4.

**FIGURE 5:**
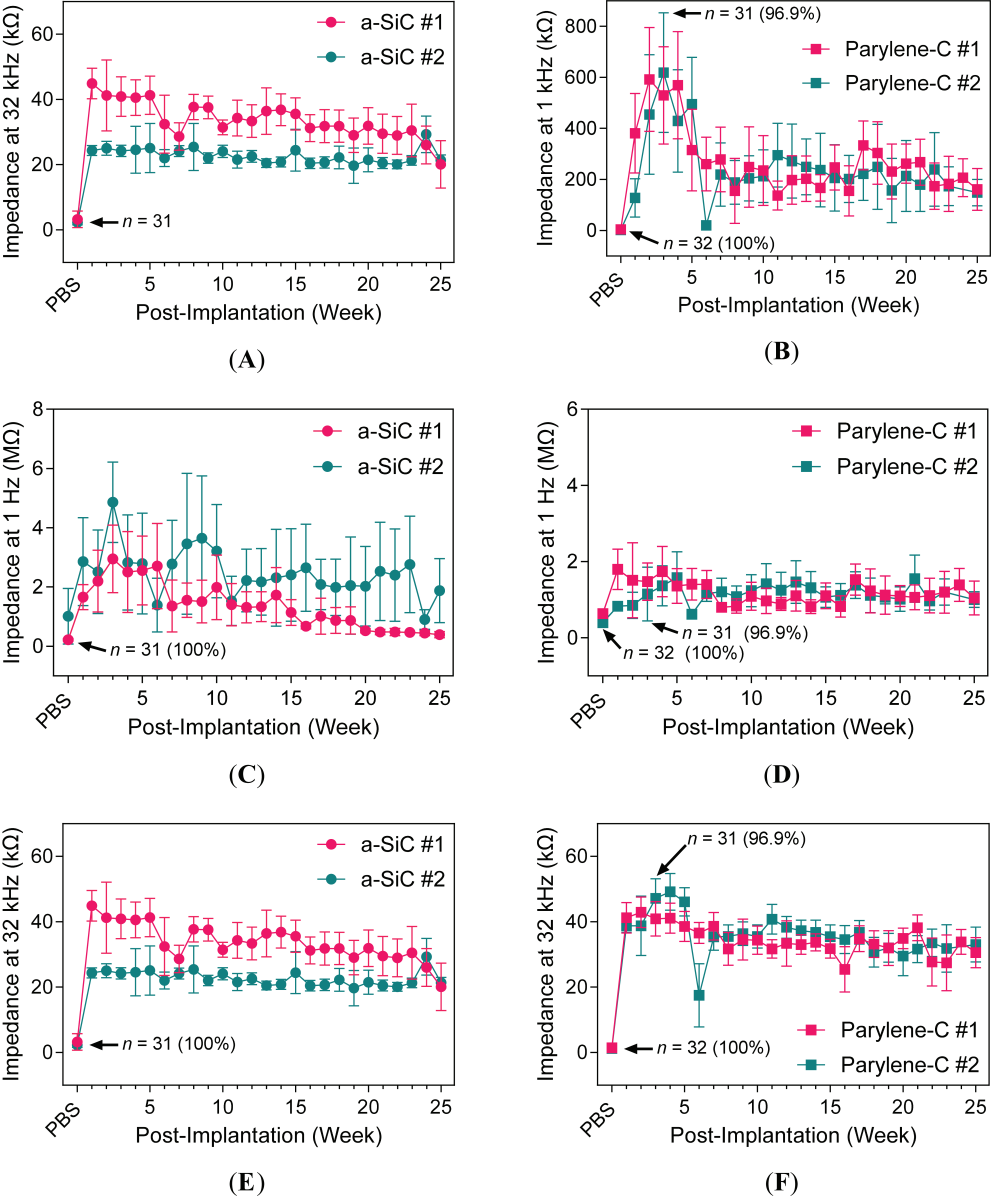
Impedance magnitude at different frequencies in PBS and rat cortex. 1-kHz impedance for **(A)** a-SiC and **(B)** Parylene-C types. 1-Hz impedance for **(C)** a-SiC and **(D)** Parylene-C types. 32-kHz impedance for **(E)** a-SiC and **(F)** Parylene-C types.

Starting at 1-week post-implantation, impedance measurements showed a dramatic increase from the prior PBS measurements, consistent with a change in tissue resistance (Cogan, 2006; Musa et al., 2009) and early inflammation and glial activation around the electrode tip (Polikov et al., 2005). The subsequent decrease in impedance after 3 weeks may indicate leakage pathway development (Black et al., 2018). The mean impedance at 1 kHz stabilized after 18 weeks for both a-SiC (Figure 5A) and Parylene-C (Figure 5B) devices. Although differences appeared intermittently between device types, both groups followed a similar trajectory toward stable 1-kHz impedance. The 1-Hz impedance for both device types, as shown in Figures 5C,D displayed some signs of leakage pathway development. The a-SiC devices exhibited decreasing impedance starting at 3 weeks, with a plateau by 25 weeks. Impedance for both device types was similar by the end of the study, converging after 16 weeks. At 32 kHz, both a-SiC (Figure 5E) and Parylene-C (Figure 5F) devices exhibited a slight decline in impedance throughout the study.

#### 3.1.2 Cyclic voltammetry

**FIGURE 6:**
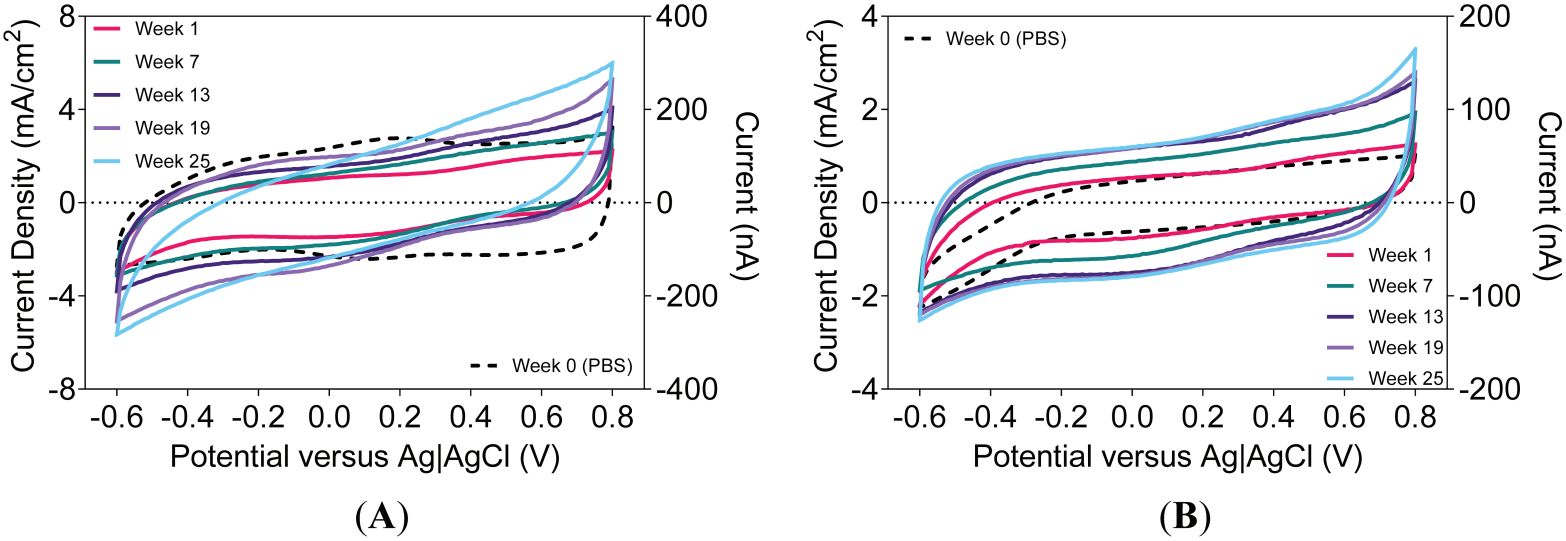
Representative cyclic voltammograms of an electrode on a UEA at 50 mV/s. Devices with **(A)** a-SiC encapsulation and UTD SIROF and **(B)** Parylene-C encapsulation and BRN SIROF. Equivalent current on right axis (1 mA/cm^2^ = 50 nA).

CVs captured at lower *v*_scan_ (e.g., 50 mV/s) can reveal encapsulation degradation, marked by increased *Q*_stor,c_ and tilted *i* − *E* curves consistent with leakage currents (Cogan, 2008). In UEAs, such changes are consistent with a breakdown of shank encapsulation or leakage through backside encapsulation or the wire bundle insulation (Black et al., 2018; Joshi-Imre et al., 2019). These measurements capture both faradaic and capacitive contributions to current. Figure 6 illustrates the tilting and broadening of CVs for both a-SiC- and Parylene-C-encapsulated devices at 50 mV/s, indicating an increase in leakage current over time.

**FIGURE 7:**
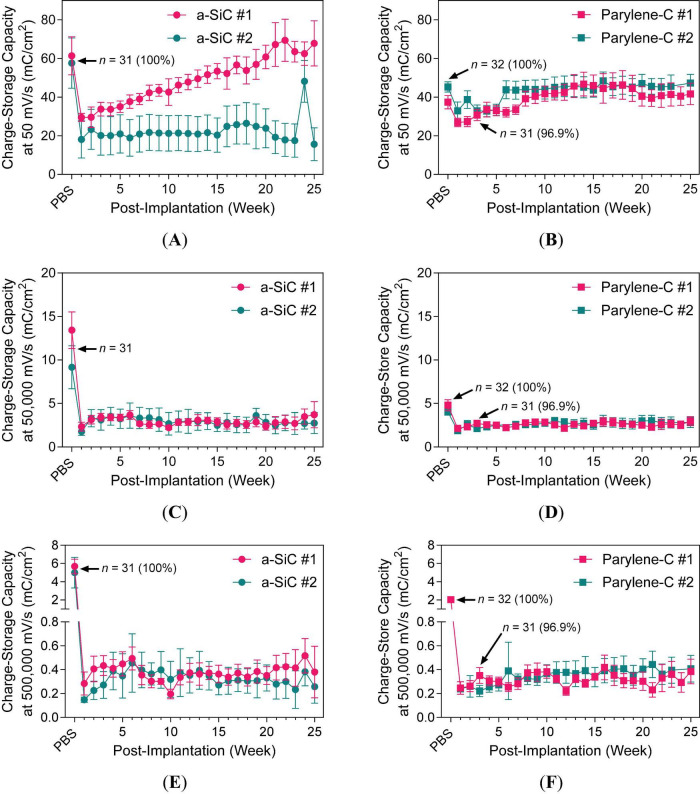
Charge-storage capacity at different scan rates in rat cortex. 50-mV/s scan rate for **(A)** a-SiC and **(B)** Parylene-C types. 50,000-mV/s scan rate for **(C)** a-SiC and **(D)** Parylene-C types. 500,000-mV/s scan rate for **(E)** a-SiC and **(F)** Parylene-C types.

Tracking *Q*_stor,c_ at 50 mV/s over time, as shown in Figures 7A,B, reveals a gradual increase followed by stabilization after approximately 17 weeks, similar to changes seen in impedance at 1 kHz (Figures 5A,B) and 1 Hz (Figures 5C,D). In Figure 7A, *Q*_stor,c_ at 50 mV/s for a-SiC array #1 increased over time and began to plateau approximately after 20 weeks, whereas array #2 remained mostly constant. CVs performed at a faster *v*_scan_, such as 50,000 mV/s, emphasize electrode surface reactions rather than leakage effects, similar in principle to higher-frequency impedance. Small increases in *Q*_stor,c_ at 50,000 mV/s (Figures 7C,D) may indicate changes in accessible conductive surface area (Cogan, 2008). Overall, there is relative stability in *Q*_stor,c_ for both device types. Both groups followed similar trajectories, converging after approximately 7 weeks. In Figures 7E,F, *Q*_stor,c_ at 500,000 mV/s remained stable throughout the study and was largely unaffected by leakage current, unlike low *v*_scan_ measurements.

### 3.2 Voltage transient measurements

#### 3.2.1 Voltage waveform characteristics

**FIGURE 8:**
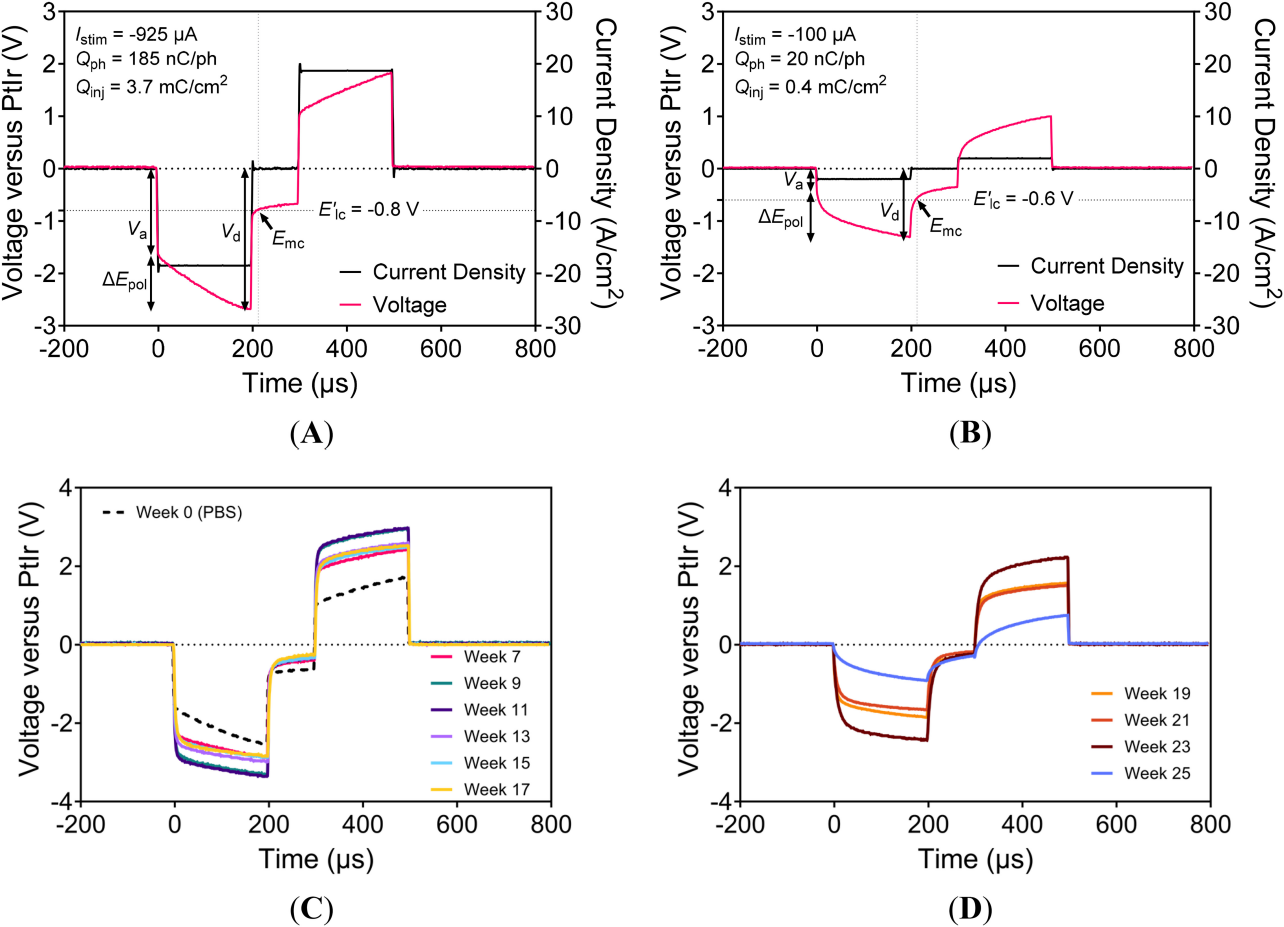
Representative voltage transient measurements at maximum *Q*_inj_ of a SIROF electrode on an a-SiC-encapsulated UEA. Measured in **(A)** PBS at 37°C with adjusted cathodic potential limit $E_{\mathrm{lc}}^{\prime }$ = −0.8 V and **(B)** rat cortex with $E_{c}^{\prime }$ = −0.6 V. *V*_a_ is the access voltage, Δ*E*_pol_ is the change in electrode polarization, and *E*_mc_ is the maximum cathodic potential excursion. The horizontal dotted line represents $E_{c}^{\prime }$, and the vertical dotted line represents the time point for locating *E*_mc_. Waveform changes **(C)** between week 0 (PBS) and 17 and **(D)** between 19 and 25. For **(A,B)**, current density scale: 1 A/cm^2^ = 50 μA = 10 nC/ph = 0.2 mC/cm^2^.

The VT waveform metrics—access voltage (*V*_a_), change in electrode polarization (Δ*E*_pol_), and driving voltage (*V*_d_)—differed notably between *in vitro* and *in vivo* environments. The *V*_a_ is the putative *i*−*R* (ohmic) drop, Δ*E*_pol_ represents the magnitude of change in electrode potential and overpotentials induced by *I*_stim_, and *V*_d_ is the voltage required to drive *I*_stim_ from the active electrode to the return electrode—measured as the potential difference between the active electrode and the return electrode. Figure 8 illustrates how VT waveforms varied between saline and rat cortex.

In PBS, as shown in Figure 8A, Δ*E*_pol_ was smaller than *V*_a_. In contrast, the *in vivo* measurement (Figure 8B) showed greater polarization compared to the ohmic drop. The cathodic potential limits were adjusted to $E_{\mathrm{lc}}^{\prime }$ = -0.8 V for PBS and $E_{\mathrm{lc}}^{\prime }$ = -0.6 V for rat cortex, based on the measured *E*_oc_ of PtIr. The VT plots were labeled with voltage since a standard reference electrode (e.g., Ag|AgCl) was not used, and the extent of PtIr polarization during pulsing remains uncertain. The changes in VT responses were observed to shrink toward the end of the study, as shown in Figures 8C,D.

#### 3.2.2 Maximum charge-injection capacity

**FIGURE 9:**
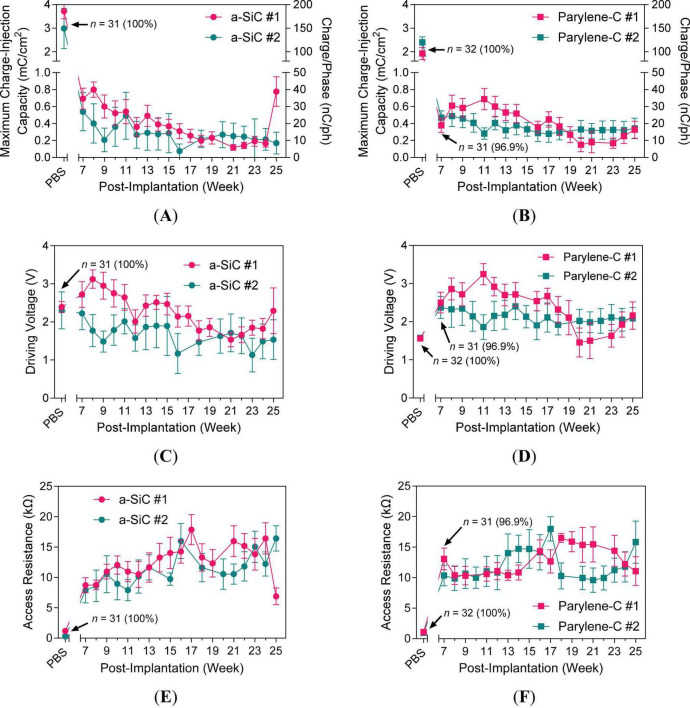
PBS and chronic voltage transient metrics at maximum charge-injection capacity. Maximum *Q*_inj_ with equivalent *Q*_ph_ on the right axis (1 mC/cm^2^ = 50 nC/ph = 250 μA = 5 A/cm^2^) for **(A)** a-SiC and **(B)** Parylene-C types. Driving voltage (*V*_d_) for **(C)** a-SiC and **(D)** Parylene-C types. Access resistance (*R*_a_) for **(E)** a-SiC and **(F)** Parylene-C types.

An 80–82% decrease in maximum *Q*_inj_ was observed for the a-SiC (Figure 9A) and Parylene-C (Figure 9B) devices from PBS to 7 weeks in rat cortex. Subsequently, the values declined over time for both device types but plateaued after about 16 weeks post-implant. The *V*_d_, shown in Figures 9C,D, gradually decreased over time, tracking with the decline in maximum *Q*_inj_. This trend in *V*_d_ demonstrated that more voltage was needed to deliver current early after implantation, but this requirement decreased as the maximum *Q*_inj_ diminished. The *R*_a_, shown in Figures 9E,F, gradually increased over time but stabilized after 17 weeks post-implant. This increase indicates that the pathway resistance between the active electrode (SIROF) and return electrode (PtIr) increased due to tissue encapsulation, which was not reflected in high-frequency impedance. The increase in *R*_a_ may relate to the reduced maximum *Q*_inj_ performance.

#### 3.2.3 Charge density comparison

**FIGURE 10:**
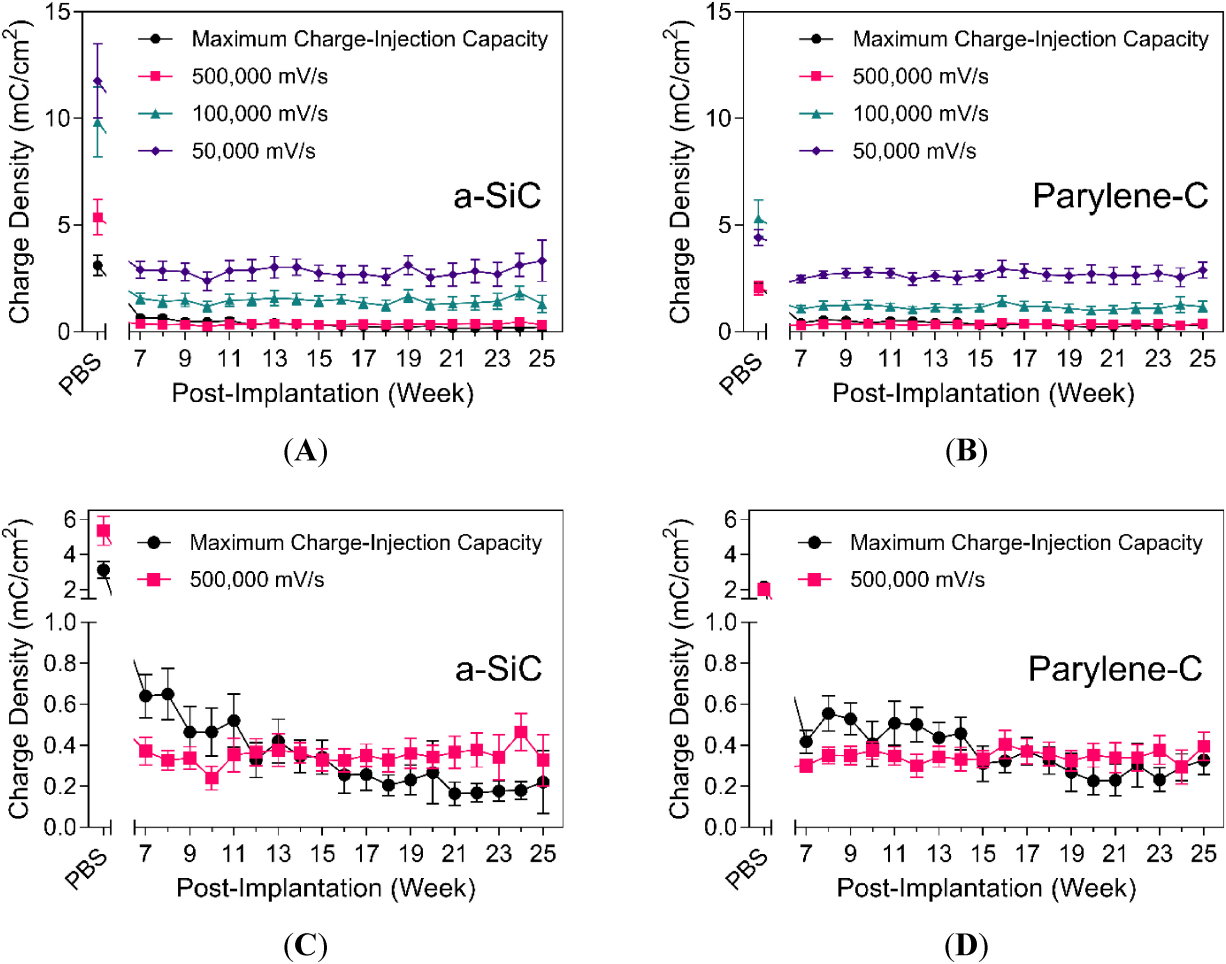
Comparison of maximum charge-injection capacity and charge-storage capacity at high scan rates. Measurements of **(A)** a-SiC devices (UTD SIROF) and **(B)** Parylene-C (BRN SIROF) devices at maximum *Q*_inj_, 50,000, 100,000, and 500,000 mV/s. In-depth comparison of **(C)** a-SiC devices (UTD SIROF) and **(D)** Parylene-C (BRN SIROF) for maximum *Q*_inj_ and 500,000 mV/s.

Comparison of charge density (mC/cm^2^) between the maximum *Q*_inj_ and various high-sweep *Q*_stor,c_ is shown in Figure 10. The PBS measurement taken prior to implantation, as shown in Figures 10A,B, shows that *Q*_stor,c_ decreases as sweep rate increases from 50,000 mV/s to 500,000 mV/s, with the maximum *Q*_inj_ from pulsing being lower than all *Q*_stor,c_ measurements. In contrast, the post-implant values reveal that the maximum *Q*_inj_ was situated between the *Q*_stor,c_ of 100,000 mV/s and 500,000 mV/s after 12–15 weeks, indicating some correlation between *Q*_stor,c_ values and maximum *Q*_inj_, at least *in vivo*. Therefore, *Q*_stor,c_ at 500,000 mV/s may serve as an alternative estimator of maximum *Q*_inj_ in rat cortex. The performance of a-SiC devices is shown in Figure 10C, and that of Parylene-C devices is shown in Figure 10D. While values varied between timepoints, *Q*_stor,c_ at 500,000 mV/s was comparable with the maximum *Q*_inj_. The similarity suggests that high scan-rate CVs can serve as a simple screening method in preclinical or device testing for estimating the maximum *Q*_inj_, while using an adequate potentiostat and careful setup.

**FIGURE 11:**
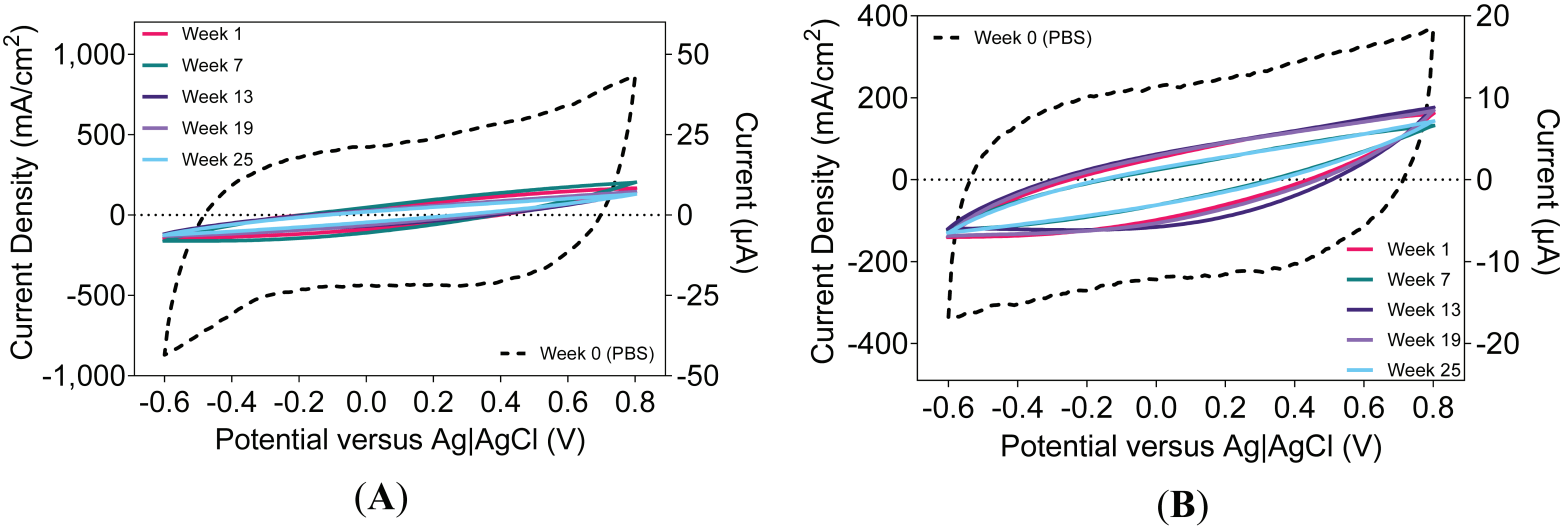
Representative cyclic voltammograms of an electrode on a UEA at 500,000 mV/s. Devices with **(A)** a-SiC encapsulation and UTD SIROF or **(B)** Parylene-C encapsulation and BRN SIROF. Equivalent current on right axis (1 mA/cm^2^ = 0.02 μA).

As shown in Figure 11, the current density of CVs at 500,000 mV/s is substantially smaller in magnitude than that encountered during VTs at maximum *Q*_inj_, being approximately 5% in maximum current density. However, both modalities exhibited similar reductions between PBS and *in vivo* measurements. With the potential range of 1.4 V, each phase of the CV at 500,000 mV/s corresponds to a duration of 2.8 ms. Despite the different orders of magnitude in current density and duration, the charge density calculated from the potential cycle is comparable to that from the current pulse. Analogous to how higher frequencies in EIS reflect more resistive elements, increasing *v*_rate_ reduces faradaic and capacitive features in *i* − *E* responses. This trend is evident in a-SiC devices (Figure 11A) and for Parylene-C devices (Figure 11B). These waveform changes likely result from both material properties and the implant environment.

#### 3.2.4 Maximum percent utilization

**FIGURE 12:**
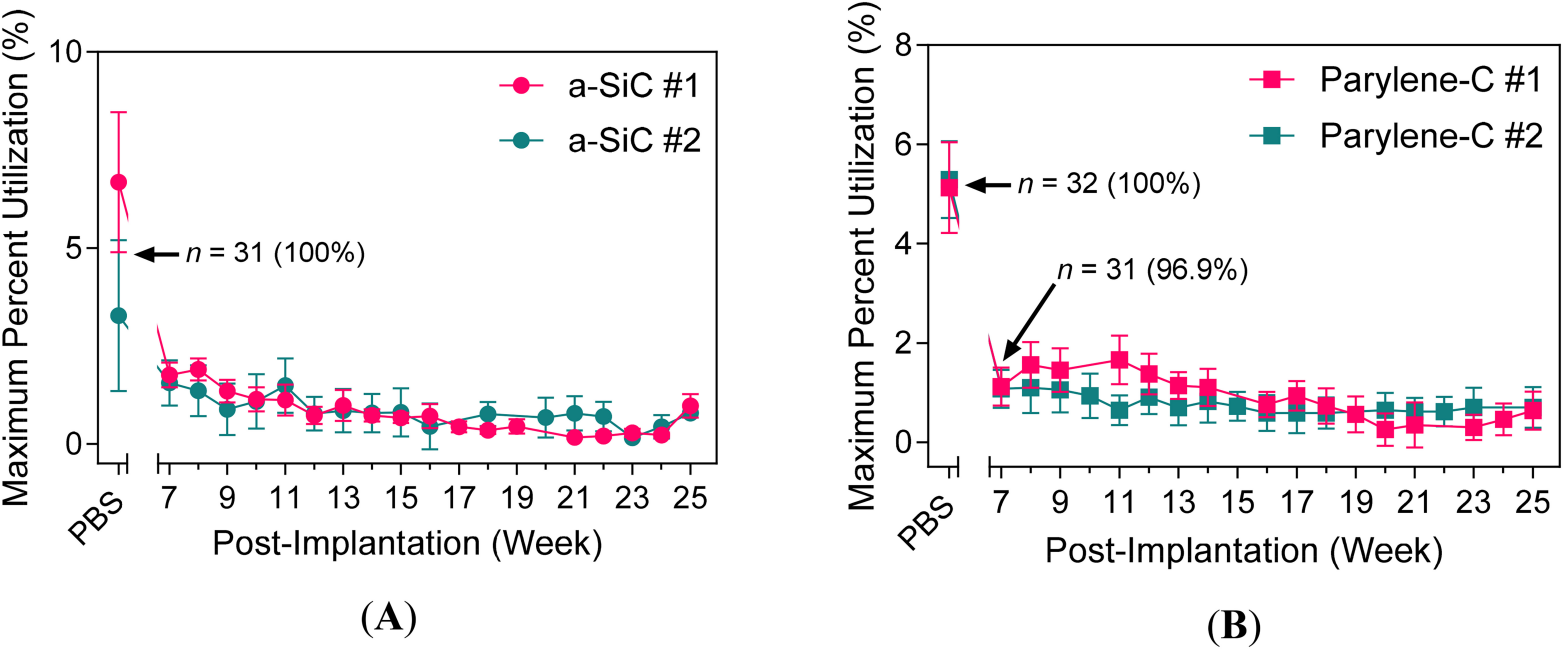
Percent utilization over 25 weeks. UEA with **(A)** a-SiC encapsulation and UTD SIROF or **(B)** Parylene-C encapsulation and BRN SIROF.

Maximum percent utilization is defined as the ratio of maximum *Q*_inj_ to *Q*_stor,c_ at 50 mV/s. This metric quantifies how much of the available stored charge is used during microstimulation (Kelliher and Rose, 1987; Frederick et al., 2020). As shown in Figure 12, the maximum percent utilization was comparable between the a-SiC (Figure 12A) and Parylene-C (Figure 12B) devices throughout the 25-week study. The similarity in percent utilization, despite differences in charge-storage and maximum charge-injection capacities, shows that both device types exhibit similar electrochemical accessibility.

## 4 Discussion

This study presents a long-term *in vivo* electrochemical characterization of Utah electrode arrays (UEAs) coated with a sputtered iridium oxide film (SIROF) and encapsulated with either amorphous silicon carbide (a-SiC) or Parylene-C over 25 weeks of intracortical microstimulation (ICMS) in rat motor cortex. To contextualize these findings relative to prior chronic UEA studies, Table 1 summarizes key experimental parameters and outcomes, including electrode area, test model, study duration, and primary measurements. Both encapsulation materials maintained functional electrochemical performance over 25 weeks.

**TABLE 1:** Comparison table of similar UEA studies.

Configuration	Model	Experiments	Key outcomes
Parylene-C encapsulation 4,000-μm^2^ SIROF electrodes (Black et al., 2018)	Rat motor cortex (24 weeks)	EIS CV VT (4 nC/ph) Single-unit recording Histology	• Single-unit active electrode yield decreased from 52.8% (week 1) to 13.4% (week 24) • Total number of single units decreased from 106 (week 1) to 15 (week 24) • EIS and CV showed changes consistent with development of leakage pathways • 10-35% electrodes were able to deliver 4 nC/ph without exceeding potential limits
a-SiC encapsulation 19,500-μm^2^ SIROF electrodes (Joshi-Imre et al., 2019)	Rat motor cortex (30 weeks)	EIS CV VT (4 nC/ph) Single-unit recording Histology	• First *in vivo* study of a-SiC on 3D and functional MEA • Active electrode yield of 94.6 ± 5.4% (week 1) decreasing to 16.4 ± 11.5% (week 30) • Impedance increase from 30.15 ± 1.69 kΩ (week 1) to 230.8 ± 79.1 kΩ (week 30) • 42.9% (week 1) to 74.2% (week 30) electrodes could deliver 4 nC/ph • Increase in *Q*_stor,c_ at slow sweep rates suggest increased conductive pathways • Variability in chronic tissue response • SIROF delamination coating and silicon corrosion on some explanted arrays
a-SiC encapsulation 19,500-μm^2^ SIROF electrodes (Nguyen et al., 2021)	PBS (255 days at 87°C)	EIS CV	• UEA with a-SiC maintained electrochemical stability for > 20 years in saline • 1-kHz impedance remained below the 70 kΩ• *Q*_stor,c_ maintained above 10 mC/cm^2^ • Increasing low-frequency impedance and decreasing high-frequency impedance may be due to leakage pathways developing from the backside of the UEA
a-SiC encapsulation 5,000-μm^2^ SIROF electrodes (Negi et al., 2024)	Rat cortex (20 weeks)	EIS CV	• UEA with a-SiC showed stable preliminary performance rat cortex • Impedance in cat nerve increased for both stimulated and unstimulated electrodes • Impedance decreased from 50 to 70% after 15 weeks in cat nerve
	Cat auditory nerve (14 weeks)	Stimulation Impedance	
a-SiC encapsulation 5,000-μm^2^ SIROF electrodes (Present Study)	Rat motor cortex (25 weeks)	EIS CV VT (Maximum *Q*_inj_)	• Chronic tracking maximum *Q*_inj_ of UEA • Automated VT enabled longitudinal maximum *Q*_inj_ determination • Alternative approach for evaluating stimulation properties • 1-kHz impedance remained stable by study end • Early transient increases in 1-Hz impedance and *Q*_stor,c_ • No sustained divergence between a-SiC and Parylene-C encapsulation

The 1-kHz impedance was stable for both materials by the end of the study. The slight decrease in impedance at 1 Hz and increasing cathodic charge-storage capacity (*Q*_stor,c_) at 50 mV/s are consistent with encapsulation degradation, likely originating from the backside silicone over the Pt-bond pads and Au-wire bundle (Black et al., 2018; Joshi-Imre et al., 2019; Woeppel et al., 2021). Medical-grade silicone (MED-4211) is used to encapsulate the wire bundle and over the bond pads for UEAs (Caldwell et al., 2017), where conductive pathways (leakage) can form between the bond pads and the silicone backing or within the wire bundle. Silicone, while known to provide long-term encapsulation, is susceptible to moisture ingress over time, causing deteriorating interface adhesion (Donaldson, 1991). One possible approach to reduce leakage may be the use of primers on silicone encapsulants used for UEAs to improve adhesion (Glogener et al., 2018). Leakage current may not prevent charge delivery, but local corrosion derived from leakage currents can lead to encapsulation failure, which can raise concerns about off-target stimulation (Dalrymple et al., 2025). Thermal accelerated aging (TAA) of UEAs with a-SiC encapsulation and SIROF electrodes has previously been reported, demonstrating the lifetime exceeding 20 years based on the Arrhenius modeling at 87°C (Nguyen et al., 2021). More recent research of a-SiC encapsulation used voltage as a mode for electrical accelerated aging (EAA)—a more relevant representation of long-term testing of electronics (Nguyen et al., 2025). Here, increasing voltage steps induced leakage currents that could be used to monitor electrochemical changes to encapsulation, and an empirical acceleration factor could be derived from varying time steps.

Histological investigations of chronically implanted UEAs have reported glial encapsulation, fibrotic sealing at the array base, and reduced neuronal density near electrode tips (Rousche and Normann, 1998; Black et al., 2018; Joshi-Imre et al., 2019). UEAs with a-SiC encapsulation in a prior study explanted devices from rat motor cortex after 30 weeks post-implantation (Joshi-Imre et al., 2019). Here, EIS measurements in model interstitial fluid showed decreased impedance at lower frequencies (<10 Hz) and increased impedance at higher frequencies (>1 kHz). It was also noted that the explanation process can cause damage to devices. Although histology was not performed in the present study, the stabilization of *Q*_inj_ and 1-kHz impedance after the early post-implant period suggests functional electrode-tissue equilibration rather than catastrophic packaging failure. Future studies combining electrochemical monitoring with systematic explant inspection and histological analysis will be required to definitively correlate leakage signatures with structural degradation.

A transient increase in low-frequency impedance and high-scan-rate *Q*_stor,c_ was observed in a-SiC devices during the early post-implant period. This may reflect early interfacial equilibration processes following implantation, including hydration dynamics and evolving electrode–tissue coupling. Structural differences between device types may also contribute, as the a-SiC devices incorporated SIROF coated over the encapsulation (Joshi-Imre et al., 2019; Nguyen et al., 2022) using O_2_ and H_2_O vapor (Maeng et al., 2019), whereas the Parylene-C devices used SIROF coated beneath the encapsulation (Bhandari et al., 2010) using O_2_ only (Negi et al., 2012). Although isolated time points showed statistically significant differences between groups, these did not persist or align across electrochemical metrics, suggesting transient fluctuations rather than sustained encapsulation-specific degradation. More recent efforts at refining EIS modeling have identified approaches that may improve the accuracy of stimulation electrode behavior (Lutz et al., 2025), which may be usefully applied in future work to elucidate the differences in electrode types *in vivo*.

The use of very high *v*_scan_ for cyclic voltammetry (CV) enables the assessment of fast electrochemical dynamics. The *Q*_stor,c_ at 500,000 mV/s was similar to the maximum charge-injection capacity (*Q*_inj_). Although potentiostats often lack the time resolution to capture meaningful transient responses, this method shows that charge-storage capacities from high-sweep CV can approximate the charge-injection properties of neural electrodes. Fast-scan CV (FSCV) is traditionally used for neurotransmitter detection at *v*_scan_ ≥ 100,000 mV/s (Rodeberg et al., 2017), but it may also be helpful for electrode evaluation. However, charging (capacitive) current scales with *v*_scan_ and can dominate faradaic current (Roberts and Sombers, 2018), and limiting the potential window can emphasize overpotentials that prevent observation of faradaic processes relevant for electrode characterization.

The automated voltage transient (VT) measurements provided a reproducible and scalable method for longitudinal *in vivo* monitoring of maximum *Q*_inj_. This study chronically tracked maximum *Q*_inj_ *in vivo* for 25 weeks through automated methods, enabling detailed evaluation of electrode performance compared to using changes in VT response at a fixed charge-per-phase (*Q*_ph_) (Black et al., 2018; Joshi-Imre et al., 2019). While chronopotentiometry measurements with a potentiostat are sometimes used to measure potential responses during constant-current pulses, potentiostats can lack pulse rate control and cannot evaluate steady-state responses under rapid pulsing conditions (Harris et al., 2019).

Behavioral effects were not observed in the animals under anesthesia for microstimulation measurements in the motor cortex. The maximum *Q*_inj_ decreased by approximately 81% from PBS to 7 weeks *in vivo* and decreased thereafter with some indication of plateauing and stabilization of maximum *Q*_inj_ after 21 weeks (Figure 9A), indicating a stabilization phase after initial tissue response. In this study, *V*_d_ decreased over time as the maximum *Q*_inj_ declined. Similarly, previous UEA studies observed decreases in *V*_d_ of UEAs with fixed 4-nC/ph testing (Black et al., 2018; Joshi-Imre et al., 2019). In our study, the access resistance (*R*_a_) gradually increased post-implant but stabilized after 17 weeks (Figure 10C). The *V*_d_ and maximum *Q*_inj_ positively correlated early in the implant period (Figure 9B) as would be expected if foreign body response affected the electrode-tissue interface. Despite gradual changes in low-frequency impedance and slow-sweep *Q*_stor,c_, 1-kHz impedance values remained within the initial order of magnitude, and electrodes retained measurable maximum *Q*_inj_ without abrupt increases in impedance or electrical discontinuity.

Electrochemical changes observed over time may reflect a combination of electrode surface evolution and packaging-related effects. Increases in *Q*_stor,c_ can indicate changes in electrochemically active surface area or hydration of SIROF, whereas reductions in *Q*_inj_ and shifts in impedance may also arise from increased *R*_a_. Low-frequency impedance reductions and increases in *Q*_stor,c_ are consistent with the development of additional conductive pathways, which may originate from backside or wire bundle silicone encapsulation or from changes at the SIROF surface itself. Notably, *Q*_stor,c_ at 50 mV/s for the a-SiC devices (Figure 7A) showed array-to-array variability, while the 1-Hz impedance did not noticeably change over time. With only two arrays per group, this divergence likely reflects array-specific differences in local tissue response and electrode–tissue coupling and may also be influenced by differences in SIROF surface state across arrays. Accordingly, results are interpreted at the device implementation level, and array-level trajectories are reported to make within-group variability explicit. Because no structural analysis was performed, these mechanisms cannot be definitively separated; however, the absence of abrupt impedance increases or loss of stimulation capability suggests progressive changes rather than catastrophic surface failure.

Regardless of the MEA type and electrode materials being studied, two-electrode setups—conventionally used in electrical stimulators—require careful selection of the return electrode, which serves as both a quasi-reference and a counter electrode. The use of Ag|AgCl as a counter electrode is complicated by instability and Ag dissolution (Seaton and Heien, 2021). Water windows reported versus Ag|AgCl must be appropriately adjusted to the quasi-reference electrode for accurate potential measurements. The observed shift in *E*_oc_ from *in vitro* to *in vivo* for PtIr alters electrode potentials, which affects the value used for potential limits during maximum *Q*_inj_ measurements. VT measurements demonstrated distinct electrochemical responses in saline and biological environments, highlighting the need to account for *E*_oc_ shifts. Quantifying these shifts using standard references over time may help to establish consistent potential limits for VTs across studies. Return electrode potential can influence two-electrode measurements by establishing a mixed-potential during the interpulse period (Merrill et al., 2005; Cogan, 2008; Nguyen et al., 2024).

Although this work focused on Utah electrode arrays, the same measurement framework—high-scan CV, VT analysis, and open-circuit potential can be applied to many other stimulation electrodes. Within the 25-week implantation period, no sustained divergence in electrochemical performance was detected between the two tested UEA implementations. It should be noted that the devices differ in SIROF deposition, fabrication process, and encapsulation stack architecture. Therefore, observed similarities or differences cannot be attributed solely to encapsulation material. Longer implantation durations will be needed to detect any differences between device types. Implantable neural electrode degradation and tissue response often progress over timelines beyond 6 months, especially Parylene-C under chronic microstimulation (Hughes et al., 2021; Woeppel et al., 2021; Bjånes et al., 2025). Extending study duration could further demonstrate the chronic stability of a-SiC and uncover cumulative biological effects on charge injection, polarization behavior, or tissue-electrode resistance.

## 5 Conclusion

Complementary approaches were introduced for evaluating the long-term stability of UEAs *in vivo*. By chronically monitoring the maximum *Q*_inj_, employing high *v*_scan_ for CVs, and automating VT measurements, this study provides a framework for assessing neural electrode performance over extended implantation periods. The presented measurement methods enable longitudinal *in vivo* tracking of electrochemical and stimulation-relevant metrics. Both tested UEA device implementations maintained functional performance over 25 weeks, although progressive electrochemical changes were observed. Observed shifts in quasi-reference potentials from *in vitro* to *in vivo* emphasize the importance of adjusting potential limits for VT measurements under physiological conditions. Longer implant durations and different stimulation modes may help clarify how encapsulation materials affect long-term charge delivery.

## Data Availability

The raw data supporting the conclusions of this article will be made available by the authors, without undue reservation.
